# Selection of Endophytic Strains for Enhanced Bacteria-Assisted Phytoremediation of Organic Pollutants Posing a Public Health Hazard

**DOI:** 10.3390/ijms22179557

**Published:** 2021-09-03

**Authors:** Magdalena Anna Karaś, Sylwia Wdowiak-Wróbel, Wojciech Sokołowski

**Affiliations:** Department of Genetics and Microbiology, Institute of Biological Sciences, Faculty of Biology and Biotechnology, Maria Curie-Skłodowska University, Akademicka 19, 20-033 Lublin, Poland; sylwia.wdowiak-wrobel@mail.umcs.pl (S.W.-W.); wojciech.sokolowski@poczta.umcs.lublin.pl (W.S.)

**Keywords:** bacteria-assisted phytoremediation, endophytes, organic pollutants, plant growth promoting bacteria (PGPB)

## Abstract

Anthropogenic activities generate a high quantity of organic pollutants, which have an impact on human health and cause adverse environmental effects. Monitoring of many hazardous contaminations is subject to legal regulations, but some substances such as therapeutic agents, personal care products, hormones, and derivatives of common organic compounds are currently not included in these regulations. Classical methods of removal of organic pollutants involve economically challenging processes. In this regard, remediation with biological agents can be an alternative. For in situ decontamination, the plant-based approach called phytoremediation can be used. However, the main disadvantages of this method are the limited accumulation capacity of plants, sensitivity to the action of high concentrations of hazardous pollutants, and no possibility of using pollutants for growth. To overcome these drawbacks and additionally increase the efficiency of the process, an integrated technology of bacteria-assisted phytoremediation is being used recently. For the system to work, it is necessary to properly select partners, especially endophytes for specific plants, based on the knowledge of their metabolic abilities and plant colonization capacity. The best approach that allows broad recognition of all relationships occurring in a complex community of endophytic bacteria and its variability under the influence of various factors can be obtained using culture-independent techniques. However, for practical application, culture-based techniques have priority.

## 1. Introduction

Human health and the health of ecosystems are inextricably linked. The deteriorating state of the environment influences the health of the human population, which is reflected in the growing number of deaths. According to the new Report No 9/2020 from the EU environment agency (EEA), already one in every eight deaths in Europe can be linked to pollution [1]. Hazardous xenobiotics are usually recalcitrant to degradation and, due to the long-range transboundary migration, can be accumulated in the environment far from the sources of their emission [2]. The scale of the problem was presented in a comprehensive review by Bartrons et al. [3]. Xenobiotics posing a public health hazard are a very diverse group of functional substances, e.g., pharmaceutical compounds, personal care products, pesticides, polycyclic aromatic hydrocarbons (PAHs), or textile dyes. Unfortunately, most of them represent persistent organic pollutants (POPs) and, despite the great removal effort, many of these substances persist in the environment as micropollutants affecting human health. The main source of their intake is inhalation with air or consumption of contaminated edible plants and meat coming from animals fed with polluted crops.

Plants may become contaminated with organic pollutants both as a result of the deposition of these compounds along with dust on the surface of leaves and through the uptake thereof with roots from the soil. The method of uptake and translocation of particular organic compounds into plant tissues depends mainly on their physical and chemical properties, e.g., molecular mass, hydrophobic/hydrophilic properties expressed as octanol—water partition coefficients (*K*_OW_), biological characteristics of the plant, and soil features [4,5,6]. Although the solubility and concentration of organic pollutants in the soil have a lower impact on the above-mentioned processes, they should be taken into account as well [5]. Globally, the principal source of POPs in vegetation in remote and rural areas is the atmosphere, while their acquisition from soil to plant roots is a secondary entrance pathway [3]. In turn, in industrialized and urbanized areas, the main source of organic contaminations in plants is preferentially the soil. 

Moderate hydrophilic substances with high solubility in water and a simultaneous ability to permeate lipid membranes are easily absorbed by plant roots and translocated to different aerial parts. The penetration of hydrophobic pollutants from the soil into plants is more complex due to their weak solubility and low bioavailability. Thanks to the active desorption mechanism with the participation of plant root exudates and binding proteins, hydrophobic contaminants are released from soil particles and can be uptaken. Since the translocation thereof is a structure-related process and takes place only for a few of them, the highest accumulation of soil hydrophobic pollutants is observed in lipophilic tissues of roots [3,4,5]. After the uptake by plants, organic pollutants may be metabolized and/or accumulated inside plant tissues or volatilized into the atmosphere. The efficiency of these processes can be enhanced with the help of plant-associated bacteria, that have the ability to transform such substances through metabolic or enzymatic processes: growth and co-metabolism, respectively [7]. Plant-associated bacteria include endophytic, phyllospheric, and rhizospheric bacteria. Endophytes seem to be the best choice for the improvement of phytoremediation.

Endophytes colonize tissues of the living plant without expressing any visible signs or symptoms. Although successful endophytic colonization is dependent on many factors, such as the host species, plant organs, geographic locality, or seasonality, when already established it is more stable than the interaction of rhizospheric bacteria with plants. Thanks to the close contact with plant cells, endophytes are able to communicate and interact with the plant more efficiently. Additionally, they do not need to compete for nutrients and the niche with the dense population of autochthonous or indigenous bacteria in the rhizosphere, and they are protected from extreme abiotic conditions. These features allow maintenance of their high abundance, which is essential for the degradation of pollutants. Moreover, unlike rhizospheric bacteria, besides the direct reduction of the content of xenobiotics inside plants and in their environment through many mechanisms, endophytes simultaneously stimulate plant defense mechanisms. Both routes counteract with abiotic stress induced by pollutions in plants. Moreover, genomic studies of endophytes have demonstrated that these microbes are far more versatile than rhizospheric bacteria and may contain genes for novel traits that are beneficial to the host plant, among them degradative ones [8,9,10]. Besides, endophytic bacteria can regulate the metabolic processes of organic contaminants in plants through horizontal gene transfer to native endophytes or to the host and gene duplication [11,12].

Since it has been suggested that the endophyte microbiome may be a subpopulation of rhizosphere-inhabiting bacteria [8], some attempts have been made to identify genomic markers of the endophytic lifestyle. Genome comparisons between bacterial endophytes and the genomes of rhizospheric plant growth-promoting bacteria indicated no definitive group of genes responsible for the colonization of plants; however, these studies are starting to unveil potential genetic factors involved in the endophytic lifestyle [8,13]. Among the bacterial genes expressed in planta and allowing colonization, two genes involved in alkane degradation are mentioned: *alkB* and *CYP153* [14]. 

Thus, the aim of our review was to find support that the synergistic use of plants tolerating high levels of contaminants and beneficial endophytic bacteria capable of degrading pollutants seems to be a promising approach for successful phytoremediation of recalcitrant organic compounds, also POPs, which have recently become a challenge.

## 2. Evidence of Benefits from the Plant-Endophyte Partnership in Proximity of Xenobiotics

Advanced treatment processes are necessary for the effective removal of organic pollutants. Some of these methods are ozonation, ultrasound, ultraviolet, Fenton processes, membrane systems, biosorption, and biodegradation both in situ and ex situ depending on environmental matrices to be treated. However, recent reports have suggested that more than one treatment technique may be required to degrade these compounds completely [15]. Thus, synergistic interactions between plants and interior plant tissue bacteria seem to be a promising approach for the effective removal of residual recalcitrant organic compounds.

The first attempts to prove the validity of this approach have already been carried out, and endophytic bacteria with the potential to be used in microbe-assisted phytoremediation have been mostly acquired from plants grown on contaminated soils [7,16,17]. Some strains able to colonize plant tissues and degrade xenobiotics were also obtained from contaminated sediments and soils [18,19] and, what is less obvious, from plants grown on non-contaminated sites [20,21]. The most commonly isolated bacterial endophytes from those niches were assigned to the genera *Pseudomonas*, *Bacillus*, *Burkholderia*, *Stenotrophomonas*, *Micrococcus*, *Pantoea*, and *Microbacterium*. They were shown to have versatile metabolic pathways for utilization of organic pollutants as the only source of carbon but more frequently and efficiently in co-metabolism, which consequently enables the microorganisms to mineralize or transform contaminants into non-toxic derivatives.

However, in order to remove contaminants effectively, partners must act synergistically. The first crucial step of the degradation of anthropogenic organic pollutants inside plants consists in the activation of aromatic rings with the participation of bacterial endophyte oxygenases followed by the action of other enzymes, e.g., esterases, reductases, or dehalogenases. In contrast, plants can increase the efficiency of the degradation by providing the bacterial partner with additional sources of carbon and nitrogen [7].

### 2.1. Removal of Hydrocarbons

Hydrocarbons comprise a broad family of aliphatic, aromatic, and polycyclic compounds with high carbon ranges. They are ubiquitous environmental pollutants generated primarily from oil spillage, pesticides, automobile oils, urban stormwater discharges, and other anthropogenic activities; nevertheless, some originate from natural sources. In some national and international documents related to risk assessment for both ecological and human exposure to petroleum hydrocarbons (PHC), the assumption that plants are unable to take up petroleum hydrocarbons from contaminated soil has appeared and, therefore, subsequent exposure at higher trophic levels is not a concern [22]. However, various studies based on chemical analyses suggest that plants are not only able to absorb PHC into their tissues, but that there is a noticeable upward trend in the hydrocarbon concentrations of the vegetation over time [3,22]. Since they are highly lipid-soluble and can be readily absorbed from the gastrointestinal tract of mammals and many of them have toxic, mutagenic, and/or carcinogenic properties, there is an urgent need to develop safe and efficient ways for removal or degradation of these contaminants [23].

It has been shown that organic contamination of soil may affect the population characteristics of endophytic bacteria [24]. For instance, in their study on bacterial community in ryegrass (*Lolium multiflorum* Lam) exposed to phenanthrene and pyrene in comparison to non-contaminated plants, Zhu et al. [25] showed that strains from the genera *Bacillus*, *Pantoea*, *Pseudomonas*, *Arthrobacter*, *Pedobacter*, and *Delftia* were present only in plants exposed to PAHs. This may suggest their potential for biodegradation of the hydrocarbons tested. Moreover, it was shown that the higher concentrations of individual or combined PAHs were accompanied by lower biodiversity of endophytes [25]. In turn, it was found in another study that inoculation of phenanthrene-contaminated wheat with PAH-degrading endophytic *Massilia* sp. Pn2 had an impact on the endophytic bacterial community structure: diversity and richness as well as the overall bacterial cell counts. Also, in this case, these relationships were associated in a contamination level-dependent manner [26]. These and similar findings may indicate the direction of further research.

Although a variety of hydrocarbon-degrading plant-associated bacteria has been isolated and characterized till now, only some of them were proved to exhibit an endophytic lifestyle. The first studies on bacterial endophytes were focused on their suitability to degrade hydrocarbons in in vitro cultures and decontaminate polluted soils. In experiments conducted by Pawlik et al. [24], more than 90% of isolates obtained from *Lotus corniculatus* L. and *Oenothera biennis* L. grown in long-term PHC-polluted sites and classified to the genera *Rhizobium*, *Pseudomonas*, *Stenotrophomonas*, and *Rhodococcus* were confirmed to be able to utilize diesel oil as a carbon source. Also, *Pseudomonas aeruginosa* L10 isolated from the roots of a reed *Phragmites australis* was shown to participate in degradation C_10_-C_26_ *n*-alkanes in diesel oil, as well as naphthalene, phenanthrene, and pyrene in individually enriched cultures. Furthermore, L10 was able to increase the petroleum hydrocarbons (PHCs) degradation rate in pot trials. These findings were confirmed by genome annotation, which indicated the presence of genes related to the *n*-alkane and aromatic compound degradation pathways in L10 [27]. The colonization of plant tissues by endophytic strains potentially involved in hydrocarbons degradation was confirmed by many other authors also with the use of PCR amplification of the following alkane-degradation genes: *alkH* (alkane hydroxylase), *alkB* (alkane monooxygenase), *c23o* (catechol-2,3-dioxygenase), *CYP153* (cytochrome P450-type alkane hydroxylase) and aromatic compound pathway genes: *pah* (alpha subunit of the PAH-ring hydroxylating dioxygenases) or *ndoB* (naphthalene dioxygenase) [16,24,28,29]. The presence of such genes was most commonly found in strains classified to *Bacillus* and *Pseudomonas* and less frequently detected in *Microbacterium*, *Rhodococcus, Curtobacterium, Pantoea,* and *Enterobacter* [14,28,29].

Compared to classical phytoremediation, the higher benefits of cooperation of endophytic strains with their host plants were observed as a higher decrease in the content of pyrene, anthracene, PHCs, or PAHs in the soil was established for *Stenotrophomonas* sp. EA1-17, *Flavobacterium* sp. EA2-30, *Pantoea* sp. EA4-40, *Pseudomonas* sp. EA6-5, *Enterobacter* sp. 12J1, *Enterobacter ludwigii* ISI10-3 and BRI10-9, *Bacillus* sp. SBER3, *Bacillus safensis* ZY16, and *Burkholderia fungorum* DBT1 [17,18,19,20,29,30]. In a similar approach, the possibility of degradation of a mixture of PAHs (naphthalene, phenanthrene, pyrene, fluoranthene) with high concentrations by endophytic *Stenotrophomonas* sp. P_1_ and *Pseudomonas* sp. P_3_ isolated from tissues of *Conyza canadensis* and *Trifolium pratense* L., respectively, was demonstrated [7]. In turn, *Paenibacillus* sp. PHE-3 isolated from *Plantago asiatica* L. exhibited an ability to degrade HMW-PAHs in the presence of other 2-, 3-ringed PAHs through co-metabolism [31]. 

Another way to improve the efficiency of hydrocarbons degradation is the use of bacterial consortia. It was shown that co-inoculation of red clover with *Rhizobium leguminosarum* and *Azospirillum brasilense* improved plant growth in conditions of PAH contamination [21].

Contrarily, little information on the reduction of PAH contamination in planta by endophytic bacteria is available. Since phenanthrene is the simplest PAH containing bay and K regions, which are found in many carcinogenic PAHs (e.g., benzo[a]pyrene), it was often used as a model substrate for studies [32]. Recently, the removal of phenanthrene from Italian ryegrass (*Lolium multiflorum* Lam) has been reported to take place with the use of two endophytic bacteria *Massilia* sp. Pn2 and *Pseudomonas* sp. Ph6 isolated from plants grown in soils contaminated with PAHs, i.e., *Alopecurus aequalis* Sobol and *Trifolium pratense* L., respectively [16,33]. The Pn2 strain was able to lower the content of phenanthrene in roots and shoots and, consequently, significantly promoted ryegrass growth observed as an increase in the fresh weight and dry weight, as well as ryegrass height and root length in a polluted environment. Furthermore, Pn2 degraded naphthalene, acenaphthene, anthracene, and pyrene in vitro, showing the potential to cope with various PAHs [16]. In subsequent studies, the subcellular distribution and the biotransformation mechanism of phenanthrene in pakchoi (*Brassica chinensis* L.) seedlings inoculated with *Pseudomonas* Ph6-*gfp* were investigated both in vitro and in vivo. The results indicated that Ph6-*gfp* colonized the pakchoi interior and reduced the content of phenanthrene in different cell compartments. The possible products of phenanthrene biotransformation were identified by high-resolution mass spectrometry coupled with ^13^C_2_-phenanthrene labeling, and three distinct reactions pathways of phenanthrene biotransformation were established (i.e., plant metabolism, endophytic degradation, and conjugation reaction). Compared with Ph6-gfp-free plants, the content of phenanthrene in inoculated leaves was 31.36–61.78% lower after 24–72 h cultivation [34]. In other studies, effective colonization of vegetable roots, including *Ipomoea aquatica* Forsk, *Brassica campestris*, and *Brassica chinensis*, with *Sphingobium* sp. RS1-*gfp* strain also led to almost complete phenanthrene reduction in roots and a considerable decline in shoots [35]. Since the performance of any single PAH-degrading strain is generally limited, more comprehensive studies were performed with a consortium of eight PAH-degrading endophytic bacteria exhibiting various properties: *Sphingobium* sp. RS1 and RS2, *Mycobacterium* sp. strains Pyr9, Phe15, and 033, *Massilia* sp. Pn2, *Paenibacillus* sp. Phe3, and *Pseudomonas* sp. Ph6 for degradation of 16 EPA priority PAHs posing a human health risk in vegetable tissues. As established, they were able to decrease the accumulation of 16 PAHs in edible parts, thereby reducing the incremental lifetime cancer risk (ILCR) values related to the consumption of contaminated vegetables [36].

However, recent data indicate that the detection and degradation of exclusively 16 apolar PAHs out of the hundreds other known is currently insufficient for prevention of the human health risk. The classical PAH watch list established in 1976 by the US-EPA does not comprise apolar PAHs containing such heteroatoms as oxygen, nitrogen, and sulfur and polar ones substituted with halogens, alkyl-, oxy-, hydroxyl-, amino-, or nitro-functional groups although many of them are believed to be more genotoxic, mutagenic, and carcinogenic [37]. The higher bioaccumulation of alkyl PAHs in comparison to their apolar homologs was demonstrated in the roots of *Echinacea purpurea* [38], which is proof that they should be taken into consideration for bioremediation in the future. However, diesel compounds such as hydroxy-PAHs and sulfur-heterocycles as well as their alkyl derivatives are very stable. Currently, only a few or no specific bacterial degraders thereof, especially endophytic ones, have been identified. Some soil bacteria obtained from petroleum-contaminated areas were found to be capable of metabolizing sulfur-heterocycle compounds. They belonged to genera often associated with the endophytic lifestyle, such as *Paenibacillus* sp. A11-2, *Sphingomonas subarctica* T7b, *Oerskovia* sp. R3 order *Actinomycetales*, and *Rhodococcus erythropolis* IGTS8 [39,40,41,42]. In turn, alkyl derivatives of benzene were degraded by *Pseudomonas stutzeri* 9 [43].

### 2.2. Decontamination of Textile Dyes

The use of dyes in textile, leather, cosmetic, pharmaceutical, and paper industries is one of the most environmentally polluting and devastating anthropogenic activities, additionally posing health hazards to humans [44]. Since they are usually water-soluble organic compounds, which can penetrate plant and animal tissues [45], the effective discharge of hazardous dyes from aqueous solutions and detoxification is crucial. There are various physical, chemical, and biological methods available for the removal of dyes from wastewater, but phytoremediation is generally considered to be the most promising and low-cost approach. Although plants play a significant role in the direct uptake of pollutants from wastewaters, the processes of transformation and mineralization of textile dyes greatly depend on microbial communities closely associated with their roots systems. Different endophytes can decontaminate textile dye wastewater through bioaccumulation, biosorption, or biotransformation, which results in not only decolorization but also detoxification of dyes in the environment. Thus, the biodegradation of textile dyes by the synergistic action of endophytes and plants seems to be a viable alternative to pure classical phytoremediation.

Textile dyes can be classified into many groups based on the structure of the chromophore. However, the most prevalent are azo dyes, anthraquinones, and triphenylmethanes. Among them, azo dyes are common xenobiotic and recalcitrant materials, due to the high stability of the azo groups (–N=N–). In order to decolorize azo dyes, it is necessary to break double chromophore bonds, but since they are very stable, their degradation with conventional physicochemical methods is usually not possible [46]. Anthraquinone dyes are the second largest class of dyes containing a fused aromatic ring structure, which makes them recalcitrant to degradation. These dyes are characterized by the presence of the chromophore group =C=O. Among triphenylmethane, crystal violet had the most stable structure due to the presence of the quaternary ammonium substituent [47].

According to the selection rule, endophytes isolated from plants growing in contaminated areas should be able to biodegrade various dyes. For example, *Exiguobacterium profundum* strain N4 obtained from *Amaranthus spinosus* collected from a site polluted with effluents from textile dyeing and printing industries was able to bleach and degrade diazo dye Reactive Black-5 by enzymatic oxidation, reduction, desulfonation, and demethylation to nontoxic benzene and naphthalene [9]. Similarly, the alkaliphilic endophyte *Bacillus fermus* (Kx898362) obtained from *Centella asiatica* showed the potential to degrade diazo dye Direct Blue-14 in in vitro assays. The disintegration patterns revealed by LC-MS showed that the parent DB-14 molecule was completely disintegrated into five noncytotoxic intermediates [46]. In turn, the endophytic bacterium *Klebsiella aerogenes* S27 obtained from the leaves of the wetland plant *Suaeda salsa* was involved in the biodegradation of triphenylmethane dye malachite green (MG) into a nontoxic metabolite N,N-dimethylaniline. The removal of MG is of great importance, since it had been extensively used in dye industries or in aquaculture as an antifungal agent before 1993 when it was nominated as a priority chemical for carcinogenicity testing by the United States Food and Drug Administration (FDA) [45].

The inoculation of PGP-endophytes to plants growing in soil irrigated with textile effluents for improvement of plant biomass production and for soil remediation is still a rare practice. Several reports are available in the literature on the bioremediation of dyes by endophytic microorganisms, mostly used in phytodepuration systems. Spectrometric analysis of the end products of degradation of sulfonated diazo dye Direct Red 5B showed that the synergistic action of the *Portulaca grandiflora* plant and *Pseudomonas putida* strain PgH resulted in higher biotransformation with enhanced efficiency than when each of them acted separately. Moreover, a phytotoxicity study revealed the non-toxic nature of metabolites formed after parent dye degradation [48]. Also, the collective action of endophytic *Microbacterium arborescens* TYSI04 isolated from shoots of *Typha domingensis* and *Bacillus pumilus* PIRI30 obtained from roots of *Pistia* enhanced textile effluent degradation and toxicity reduction, which was confirmed by significant reductions in chemical oxygen demand—COD (79%), biological oxygen demand—BOD (77%), total dissolved solids—TDS (59%), TSS (27%), and color removal within 72 h when a combination of plants and bacteria was applied [49]. A similar effect was achieved by Nawaz et al. [50] with the use of a consortium consisting of PGP strains (i.e., *Acinetobacter junii* NT-15, *Rhodococcus* sp. NT-39, endophytic *Pseudomonas indoloxydans* NT-38), and *Phragmites australis* for removal of three commonly used acid metal textile dyes containing two sulfo groups: Bemaplex Navy Blue D-RD, Rubine D-B, and Black D-RKP Bezma from water. Based on in vitro and in vivo characterization, in terms of Reactive Black 5 decolorization activity, a consortium of strains *Pseudomonas fluorescens* CWMP-8R25, *Microbacterium oxydans* CWMP-8R34, *Microbacterium maritypicum* CWMP-8R67, *Flavobacterium johnsoniae* CWMP-8R71, *Lysinibacillus fusiformis* CWMP-8R75, and *Enterobacter ludwigii* CWMP-8R78 isolated from *P. australis* was identified as promising in phytodepuration systems. The *F. johnsoniae* and *E. ludwigii* strains also decolorized Bezactive rouge S-Matrix, Tubantin blue, and Blue S-2G in an in vitro assay [51].

Some endophytic bacteria possess biosorption and bioaccumulation properties that can be exploited in dye decontamination. However, the bioaccumulation process is usually not preferred, compared to biosorption, because the live microbial biomass requires nutrients and supplements for its metabolic activities, which in turn would increase BOD or COD in the aquatic environment. In turn, biosorption via mechanisms such as adsorption, absorption, ion exchange, precipitation, and surface complexation needs large amounts of biomass, which is economically and technologically unfavorable. Thus, the main mechanism of biotransformation of pollutant dyes by bacterial endophytes takes place through the action of highly oxidative and non-specific ligninolytic enzymes: laccase, azo reductase, peroxidases, tyrosinase, and hydrogenase [52]. In the azoreductase-mediated cleavage of the azo bonds, toxic aromatic amines are released, which next need to be transformed into non-toxic compounds. Moreover, azo reductases are oxygen-sensitive and degrade azo dyes only in the presence of reducing equivalents FADH and NADH anaerobically. Unlike peroxidases, laccases oxidizing a wide range of polyphenols, methoxy-substituted phenols, and diamines do not produce toxic peroxide intermediates from azo dyes. Bioinformatic analysis carried out by Ausek et al. [53] revealed a high diversity of genes for laccase-like enzymes among diverse bacteria, including the most common endophytic genera *Streptomycetes*, *Bacilli*, and *Pseudomonads* as well as anaerobes, autotrophs, and alkaliphiles. Additionally, most of them had signal peptides indicating that these laccases may be exported from the cytoplasm, which improves their potential for future biotechnological application. However, only a few of them were detected in strains obtained from plant tissues. It was shown that the endophytic bacterium *Pantoea ananatis* Sd-1 isolated from rice seeds produced both intra- and extra-cellular laccases, of which extracellular Lac4 exhibited degradation of non-phenolic and phenolic compounds and decolorization of various synthetic dyes (azo dye Congo Red, anthraquinone dye Remazol Brillant Blue-R, and dyes from the group of triphenylmethane Aniline Blue) [54]. The laccase gene and activity was also confirmed in the *Sinorhizobum meliloti* strain L3.8 isolated from root nodules of *Medicago* sp. [55]. Another extracellular oxidoreductase enzyme triphenylmethane reductase-like (TMR-like) was involved in the biodegradation of malachite green by endophytic *Klebsiella aerogenes* S27. Since there is no report on plants harboring *tmr* genes, bacteria possessing the gene could be very valuable in endophyte-assisted phytoremediation [45].

### 2.3. Bioremediation of Polyhalogenated Organic Compounds—Biphenyls and Dibenzodioxins

Polychlorinated biphenyls (PCBs) are classified as POPs with high toxicity. Many of these pollutants, among them bisphenol A (BPA), are recognized as endocrine-disrupting compounds (EDCs) due to their ability to interfere with the human endocrine system. At low concentrations, BPA can also show acute toxicity toward aquatic organisms and carcinogenic properties [56]. In turn, members of the family of polychlorinated dibenzodioxins (PCDDs) can bioaccumulate in humans and wildlife due to their lipophilic properties and may cause developmental disturbances and cancer. The European Union Water Framework Directive [57] and the Directive of the European Parliament and Council (2013/39/EU) regarding priority substances in the field of water policy (Directive EQS) list 45 substances representing a serious threat to aquatic environments and to humans, which need to be removed from aquatic environments, including PCBs and PCDDs. 

Recently, the potential for improvement of removal of BPA in planta has been shown by endophytic *Pantoea anantis* in combination with its host plant *Dracaena sanderiana*. Due to the activities of the plants and microorganisms, such physicochemical indicator parameters as pH, COD, BOD, TDS, conductivity, and salinity were reduced after 5 days of the experimental period with a decrease in BPA levels [56,58]. Bioremediation of the most toxic dioxin congener 2,3,7,8-TCDD was shown in a study involving the endophytic bacterium *Burkholderia cenocapacia* 869T2 isolated from roots of vetiver grass. In an in vitro assay, it was capable of TCDD degradation by nearly 95% after one week of aerobic incubation. Generally, in the bioremediation of dioxins by bacteria, angular dioxygenase, cytochrome P450, lignin peroxidase, and dehalogenases are known as important dioxin-metabolizing enzymes. Through transcriptomic analysis of strain 869T2 exposed to TCDD, a number of catabolic genes involved in dioxin metabolism were detected with high gene expressions in the presence of TCDD. Assays with cloned l-2-haloacid dehalogenase (2-HAD) indicated that it might play a pivotal role in TCDD dehalogenation [59].

### 2.4. Removal of Agrochemicals—Pesticides/Herbicides/Insecticides/Fertilizers

Despite some positive impact of the use of herbicides, pesticides, and insecticides on an increase in crop production, there are reports on many negative effects of their use such as selection for resistant weeds, production of toxic metabolites from their degradation, changes in soil microbial communities and biogeochemical cycles, alterations in plant nutrition and soil fertility, and persistent environmental contamination. The chemical structures of active ingredients present in such herbicide formulations, including oxygen, hydroxide, sulfonyl, phosphoric acid, amine, and chlorine, differentially affect environmental matrices and many non-target plant and animal organisms, including humans [60]. Although such commonly used pesticides as 2,4-dichlorophenoxyacetic acid (2,4-D) and atrazine are not listed by the Stockholm Convention as POPs, they have been listed by the US-EPA as toxic and are associated with human health risks.

To overcome these limitations and mitigate their impact, some endophytic bacteria were used for the transformation of these substances via xenobiotic degradation pathways. For example, an endophytic *B. megaterium* strain obtained from the roots of tobacco degraded 93% of quinclorac, i.e., a herbicide used to control several grass species in rice, canola, barley, corn, and sorghum, and alleviated its phytotoxicity [61]. Detoxification of atrazine, which is recognized as a major contaminant of surface and groundwater, by endophytic *Streptomyces* sp. isolated from sugarcane was confirmed in a study conducted by Mesquini et al. [62]. The usefulness of bacterial endophytes to enhance the phytoremediation of herbicide residues in crop plants was shown in a system of *Pisum sativum* with endophytic *Pseudomonas putida* strain VM1441, which naturally possesses the ability to degrade 2,4-D [63]. 2,4-D is one of the most commonly used and potentially health-threatening herbicides in the world. Despite its rapid spontaneous degradation, the excessive use of the herbicide leads to its significant accumulation in the environment, which cannot be removed by plants that are typically used for phytoremediation due to their limited capacity. Thus, as demonstrated above, a solution to this problem may be the use of bacterial enhanced phytoremediation. Another common group of toxic pesticides with high stability in nature, which leads to their bioaccumulation, are organochlorine pesticides (OCPs) such as aldrin, dieldrin, dichlorodiphenyltrichloroethane (DDT), benzene hexachloride (BHC), pentachloronitrobenzene (PHNB), and hexachlorocyclohexane. Chlorpyrifos (CP), i.e., an insecticide exerting adverse effects on animals and plants represents OCPs as well. Enhanced CP removal from ryegrass (*Lolium multiflorum*) hosting the endophytic *Mezorhizobium* sp. HN3 was reported by Jabeen et al. [64].

An important activity offered by endophytic bacteria may also be associated with the reduction of the content of herbicide residues in food plants and herbs. In this context, endophytic bacteria *Sphingomonas* sp. HJY isolated from Chinese chives were shown to lower the content of CP in host plants and in liquid medium in an in vitro study [65,66]. Likewise, in vitro and in vivo assays corroborated the simultaneous degradation of fluazinam, BHC, PCNB, CP, and DDT into non-toxic alkanes in ginseng roots, stems, and leaves by endophytic *Paenibacillus polymyxa* [67]. In similar studies, a consortium of five PGP endophytic bacteria identified as *Pseudomonas aeruginosa* strain RRA, *Bacillus megaterium* strain RRB, *Sphingobacterium siyangensis* strain RSA, *Stenotrophomonas pavanii* strain RSB, and *Curtobacterium plantarum* strain RSC, enhanced degradation of CP in rice plants and grains [68]. The mechanism of CP degradation was shown for endophytic *Pseudomonas* sp. BF1-3 was obtained from the roots of the balloon flower (*Platycodon grandifloras*). It appeared that the strain degraded CP with organophosphorus hydrolase, which was confirmed after cloning the *ophB* gene into *E. coli* [69].

Pyrethroids are highly toxic and persistent broad-spectrum pesticides mainly used against agricultural and household pests. Cypermethrin, cyhalothrin, deltamethrin, cyfluthrin, and bifenthrin are common examples of synthetic pyrethroids. In a huge number of reports, the soil bacterium *Bacillus thuringiensis* has been shown to be a multifaceted microorganism. Although the soil is its natural resource, some studies on cabbage, cotton, legumes (soybean, rice bean, French bean, lentil, and pea), and medicinal plants (Indian Ginseng and greater celandine) reported that *B. thuringiensis* was also successful in endophytic colonization [70]. Moreover, it has recently been shown to be engaged in biodegradation of cypermethrin, a man-made insecticide with adverse effects exerted particularly in sensitive populations such as aquatic organisms, and cyhalothrin [71,72]. This all indicates that the bacterium has potential as a bioinsecticide endophyte with xenobiotic removal capabilities. 

### 2.5. Removal of Pharmaceutical and Personal Care Products (PPCPs)

Pharmaceuticals are biologically active compounds used in the treatment of humans and animals, while personal care products are chemicals used for hair, skin, and dental care such as dyes, ultraviolet (UV) blockers, fragrances, and preservatives. The sources of PPCP residues that enter surface waters and wastewater are the pharmaceutical industry, ineffective disposal, and waste from hospitals and households. Frequently, expired or unused drugs are disposed of in toilets and then enter municipal wastewater. This exacerbates the influx of various pollutants in the environment affecting living organisms through multiple routes. Currently, analgesics and nonsteroidal anti-inflammatory drugs (NSAIDs) are classified as one of the most emerging groups of xenobiotics and have been detected in various natural matrices [73]. Furthermore, hormonal agents, psychotropic drugs, anti-cholesterol drugs, and β-blockers have also been detected in samples from surface water, groundwater, and treated sewage. At present, wastewater treatment plants are not required to check the effectiveness of treatment in terms of pharmaceutical content. The European Union and US-EPA are working on introducing regulations of the maximum allowable concentration values for some pharmaceuticals, e.g., diclofenac, estriol, 17-β-estradiol, and 17-α-ethinylestradiol, which were already included in Directive 2013/39/EU in the so-called “watch list” [57]. Over 90% of PPCPs remain in activated sludge in WWTPs [74]. Much of the rest could be removed with other conventional water treatment techniques such as biological oxidation/biodegradation, coagulation/flocculation, ozonation, electrodialysis, reverse osmosis, sedimentation, filtration, activated carbon, and phytoremediation. Due to the limitations of the methods, many of these contaminants remain in aquatic matrices as hazardous micropollutants, e.g., EDCs. Thus, biotechnological approaches for targeted inoculation of plant species utilized in phytoremediation might boost and improve the performance of secondary or tertiary treatment in wastewater treatment. 

Although the removal of PPCPs by constructed wetlands has been extensively studied, information on plant-microbe interaction in their biodegradation remains limited to date. By employing 16S rRNA amplicon sequencing, Sauvêtre et al. [75] showed that, in the microbiome of *Miscanthus × giganteus* exposed to diclofenac and sulfamethoxazole, *Actinobacteria* from the genera *Streptomyces*, *Microbacterium*, and *Glycomyces* were more abundant than in non-treated plants. Among cultivable endophytic isolates, *Streptomyces griseorubiginosus* strains DS24 and DS4 and *Microbacterium* sp. MG7 obtained from *Phalaris arundinacea* and *Miscanthus × giganteus* roots, respectively, were indicated as the most promising in the degradation of these PPCPs. Since the isolates showed moderate activity in in vitro assays in both cases, they should be used in consortia rather than individually [75,76]. Despite the increase in the interest in the degradation of non-steroidal anti-inflammatory drugs, little is known about the microbiological breakdown of naproxen. The presence of two condensed rings provides the relatively high stability of the molecule and its resistance to microbial degradation. To date, only a few bacterial strains possessing enzymes of the full naproxen degradation pathway have been described: *Planococcus* sp. S5, *Bacillus thuringiensis* B1(2015b), *Stenotrophomonas maltophilia* KB2, and *Pseudoxanthomonas* sp. DIN-3, but none of them were isolated from plants. Among them, *B. thuringiensis* B1(2015b) was also able to degrade ibuprofen [77,78]. 

In the environment, there are increasing amounts of EDCs that disrupt the work of hormones. This problem is relatively new and currently raises concerns. Oxybenzone (hydroxybenzophenone-3; BP3) is one of such compounds and, what is worse, the substance is consciously introduced into the environment in a way that allows it to quickly enter the body. Oxybenzone is an organic component used in sunscreens, which the FDA placed on a list of the 12 hazardous filters in 2019 [79]. Its action was found to be especially dangerous for future mothers and responsible for the underdevelopment of the fetus. However, it seems that BP3 may exert a negative impact on all users since it was detected in the human organism even after a single use and it generates large amounts of free radicals. Since its removal by WWTPs is insufficient, the potential decontaminationand degradation of BP3 by phytotreatment using hydroponic cultures of *Cyperus alternifolius* and a hairy root culture of *Armoracia rusticana* was investigated. In both studies, the elimination of the contaminant from the medium, although to some limited extent, was confirmed [80,81]. Since no endophytic bacteria capable of transformation/degradation of BP3 have been reported to date, no research on bacteria-associated phytoremediation of BP3 are available. However, the isolation of *Sphingomonas wittichii* strain BP14P from WWTP sludge utilizing BP3 as the sole energy and carbon source and completely degrading it within seven days gives hope for the future [82]. Similarly, bacteria capable of 17β-estradiol, estrone, and estriol degradation have been isolated from various environmental systems, such as wastewater, compost, constructed wetlands, and activated sludge, but not from plant tissues. They included phylogenetically diverse groups *Proteobacteria*, *Actinobacteria*, *Bacilli*, and *Flavobacteria* and species/genera typical for the endophytic lifestyle: *Stenotrophomonas maltophilia*, *Novosphingobium*, *Pseudomonas*, *Bacillus*, and *Acinetobacter*, *Rhodococcus* [83,84].

The available research on the metabolism of pharmaceuticals by endophytic microorganisms addresses mainly in vitro degradation in synthetic culture media without the host plant. However, it was established that endophytes of *P. australis*, i.e., *Rhizobium radiobacter* Cb58 and *Chriseobacterium nitroreducens* Cb55, improved the removal of the antiepileptic drug carbamazepine performed by horseradish by 21 and 10%, respectively, in simultaneous cultures with the host plant [85]. In a previous study, they were also shown to support the host plant in xenobiotic stress conditions by promoting its growth and/or degrading the parent compound directly [86].

## 3. Indirect Methods Improving the Efficiency of Bacteria-Assisted Phytoremediation 

In order to be suitable for phytoremediation, plants need to be adapted to the polluted environment. Such plants, called hyperaccumulators, should remove, absorb, assimilate, or metabolize hazardous materials from the environment with high efficiency. The activation of enzymatic systems capable of transformation, degradation, removal, and changing the bioavailability of xenobiotics takes place when contaminants exceed a certain level in plant tissues, which results in a reduction of their content, thereby preventing plant damage [34]. However, the excessive accumulation of pollutants inside plants can cause changes often leading to plant poisoning, which manifests itself in abnormal cell ultrastructure, disturbed cell biosynthesis with especially detrimental effects on photosynthesis and the synthesis of proteins, amino acids, nucleic acids, lipids, and hormones, DNA fragmentation, membrane integrity, fluidity, and permeability. Moreover, the metabolism of xenobiotics often requires mobilization of the entire energy potential of the cell leading to reduction of plant growth and subsequent phytoremediation efficiency. It is also reflected by the ability of the defense system to resist attacks by phytopathogens [5,21]. Thanks to the development of transcriptomics and proteomics, it has been demonstrated that xenobiotic effects may involve not only biochemical and physiological disruption mainly related to oxidative stress but also the disruption of signaling pathways [87]. 

Some studies have revealed that PAHs, PCBs, and herbicides inhibit photosynthesis. PAHs can cause DNA damage, especially in leaves, while chlortetracycline results in inhibition of mitotic division in plant root tip cells probably by depression of protein synthesis and enzyme activities [5,88].

Therefore, to overcome contaminant-induced stress responses and boost plant growth, thereby indirectly increasing the efficiency of phytoremediation, some beneficial PGP endophytic bacteria may be used. PGP endophytes, even lacking catabolic genes, may contribute to the alleviation of the phytotoxicity of organic pollutants via induction of defense mechanisms against phytopathogens and/or directly through solubilization of mineral nutrients (nitrogen, phosphate, potassium, iron, etc.), production of plant growth promoting substances (e.g., phytohormones), and secretion of specific enzymes (e.g., 1-aminocyclopropane-1-carboxylate deaminase; ACC deaminase). An example of such an effect is the interactions between *Burkholderia* sp. strain PsJN with two different trees *Acacia ampliceps* and *Eucalyptus camaldulensis*, which were grown for one year on soil irrigated with secondary treated textile wastewater. The combined use of plants and ACC deaminase producing PsJN resulted in enhanced production of biomass up to 12% and chlorophyll content in comparison to textile dye-stressed plants, which resulted in 29% higher soil cleaning. These results indicated the significance of the ACC enzyme of the PsJN strain in augmenting the survivability and sustainability of the host plants in harsh environmental conditions and the improved soil bacteria-assisted phytoremediation [52,89]. An example of a different protection mechanism can be observed in phenanthrene-stressed pakchoi inoculated with *Pseudomonas* Ph6-*gfp*. After successful colonization of host tissues, the endophytic strain hindered the subcellular distribution of phenanthrene from the intercellular space to subcellular fractions (i.e., cell wall, cell membrane, cell solution, and cell organelles), likely resulting from the interception and biodegradation of phenanthrene by the bacterium between the cell wall and intercellular space, which prevented cytotoxicity-induced cell degradation [34].

For simultaneous action with phytoplants, isolates that combine PGP features and contain appropriate catabolic genes are the most eligible. It was demonstrated that endophytic *B. subtilis* ZY16 able to degrade hydrocarbons showed parallel plant-growth-promoting attributes, such as siderophore and indole-3-acetic acid production, as well as phosphate solubilization. Results from a mass balance study of ZY16-inoculated *C. virgata* Sw. grown in oil-polluted saline soil confirmed the significant enhancement of both aerial and underground biomass of the ZY16-inoculated plants and improvement of decontamination of soil via direct and indirect mechanisms [17]. Other examples of the impact of PGP-endophytic bacteria on augmented xenobiotic phytoremediation are included in Table 1.

### Mechanisms of Reduction of the Impact of Xenobiotic-Induced Stress Conditions by Bacterial Endophytes

**Figure 1:**
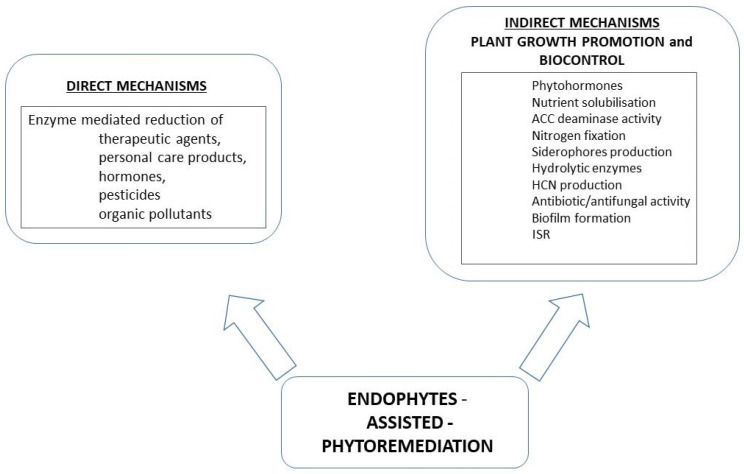
Possible mechanisms of boosting phytoremediation with participation of bacterial endophytes.

PGPB have developed a number of mechanisms positively influencing the growth and development of plants. These mechanisms can be divided into two groups: direct and indirect (Figure 1). Microorganisms belonging to PGPB can have a positive effect on plants by increasing the available forms of nitrogen, phosphorus, and iron or by producing active substances that inhibit the growth of plant pathogens or kill phytopathogens [90].

Research on this group of bacteria is often carried out in laboratory conditions and is focused on, e.g., the ability to synthesize phytohormones. The synthesis of phytohormones is one of the direct mechanisms promoting plant growth and development. Through the synthesis of phytohormones, microorganisms increase the endogenous pool of growth regulators in the plant: cytokines, gibberellins, and auxins. An important group of phytohormones produced by PGPB are auxins, including indole-3-acetic acid (IAA). Auxins regulate the processes of cell growth, fruit ripening, rooting, or aging in plants. It has also been shown that IAA can be a signal molecule in the Quorum Sensing (QS) process in bacteria that are involved in biofilm formation. The production of IAA by endophytic microorganisms is related to the presence of its precursor (most often L-tryptophan) in the root exudate. There are differences in the modes of IAA synthesis in bacteria that favor plants and in pathogens. The first group most often uses the indole-3-pyruvic acid pathway (IPyA indole-3-pyruvate pathway), while phytopathogens produce IAA from indole-3-acetamide (IAM indole-3-acetamide pathway) [90,91].

ACC deaminase synthesis is an example of a direct growth promotion mechanism by PGPB. This enzyme degrades the ethylene precursor 1-aminocyclopropane-1-carboxylic acid (ACC). As a result of this process, the concentration of ethylene in the plant decreases. This hormone is produced under biotic and abiotic stress and, in high concentrations, can damage or kill plants. The ACC deaminase enzyme hydrolyzes 1-aminocyclopropane-1-carboxylic acid, thus contributing to lowering the level of ethylene and protecting the plant against the negative effects of this hormone [92,93]. In addition, the ammonia formed as a result of ACC hydrolysis can serve as a source of nitrogen for bacteria, especially when they live on soils poor in this element. Among the microorganisms showing the ability to synthesize ACC deaminase, bacteria representing the species *Rhizobium, Pseudomonas, Burkholderia, Bacillus, Ochrobactrum,* etc. should be mentioned [94]. Some researchers postulated that the activity of deaminase ACC is the main plant growth-promoting factor of endophytic strains. Moreover, it should be noted that strains with the ACC deaminase ability often have more plant-beneficial traits such as siderophore and IAA production, etc. [92,94].

An important factor influencing the proper growth and development of plants is phosphorus. Its deficiency affects, among others, the course of energy processes and the photosynthesis process in the plant, the development of the root system, plant resistance to stress factors, or the content and quality of fats, proteins, and carbohydrates in the plant. In soil, phosphorus can form sparingly soluble salts with metal cations—Al^3+^, Ca^2+^, Fe^3+^ or Mg^2+^. Consequently, it becomes inaccessible, leading to phosphorus starvation in the plant. Some soil microorganisms show the ability to solubilize phosphorus by secreting various organic acids, e.g., acetic, citric, or succinic acid [95,96]. The process can also be based on the chelation of phosphorus-bound cations. Another mechanism is related to the mineralization of organic phosphorus forms. In this case, the microorganisms produce acid phosphatases, which convert organic phosphorus into inorganic forms, and phytase, which degrades phytates, i.e., the main source of organic phosphorus [97].

Iron is an important micronutrient necessary for the proper development and functioning of both microorganisms and their plant host. The presence of this element determines the course of such life processes as respiration, photosynthesis, chlorophyll biosynthesis, or the process of biological nitrogen fixation [97]. Siderophores are low-molecular non-protein compounds showing a high affinity and the ability to chelate iron Fe (III). Due to these properties, at low iron content in the soil, PGPB are more competitive with microorganisms that do not have the ability to synthesize siderophores. Additionally, the production of siderophores stimulates plant development by inhibiting the development of phytopathogens. This is caused by a reduction of the amount of available iron, which is necessary for the proper functioning of pathogens. This phenomenon has been described, e.g., for maize endophytes. Strains representing the genus *Bacillus* and capable of synthesizing siderophores inhibited the growth of fungal pathogens such as *Fusarium verticillioides* or *Colletotrichum graminicola* [97,98,99]. Another example is the *Pseudomonas aeruginosa* 7NSK2 strain. It produces two types of siderophores, i.e., pyoverdine and pyochelin [100]. The production of pyochelins is associated with the protection of plants against phytopathogens. Studies have shown that *Pseudomonas aeruginosa* 7NSK2 limited the growth of *Pythium splendens*, a root rot fungus in tomatoes [101]. In addition to iron, siderophores have the ability to chelate other metals, such as Zn, Ni, Cu, Cd, and others, which have been used in the phytoremediation process. Siderophores produced by PGPB also show the ability to trigger induced systemic resistance in plants [102]. 

Nitrogen is an important nutrient necessary for the proper functioning of organisms. Despite its high availability in the environment, atmospheric nitrogen is not absorbed by plants until it is reduced to ammonia. The ability to fix gaseous nitrogen biologically (BNF) is demonstrated by some microorganisms representing PGPB. This group of organisms includes free-living N2 assimilators (e.g., *Azotobacter, Clostridium*), associative microorganisms such as *Azospirillum*, and atmospheric nitrogen-fixing microorganisms in symbiotic systems (e.g., *Rhizobium, Bradyrhizobium, Frankia*) [103]. All these microorganisms showing the ability to bind atmospheric nitrogen, carry out the process of reduction of the element with the participation of an enzyme system, the most important component of which is the nitrogenase enzyme. It should be emphasized that the biological nitrogen fixation process is one of the main mechanisms used by PGPB to promote plant growth [97,104]. 

The indirect mechanisms of plant growth promotion by PGPB include the synthesis of hydrogen cyanide (HCN), the production of hydrolytic enzymes, the synthesis of antibiotics, polysaccharides, or induction of induced systemic resistance (ISR) [90,105]. Some representatives of PGPB use the synthesis of hydrogen cyanide (HCN) in plant biocontrol. The toxic effect of HCN is related to its ability to inhibit cytochrome c oxidase as well as other important metalloenzymes. It should be noted that HCN-producing microorganisms often have other biocontrol mechanisms as well. It was found that the production of HCN by PGPB may improve the effectiveness of antibiotics synthesized by these microorganisms or enzymes that degrade the cell wall of phytopathogens [106].

PGPB bacteria have the ability to synthesize many lytic enzymes hydrolyzing the cell wall of phytopathogens. The enzymes synthesized by PGPB include β-1,3-glucanase, chitinase, cellulose, and protease. The ability to synthesize β-1,3-glucanase was found in e.g., *Paenibacillus* and *Streptomyces* strains. The enzyme produced by these bacteria degraded the cell wall of fungal phytopathogens: *Fusarium oxysporum, Rhizoctonia solani, Sclerotium rolfsii*, and *Pythium ultimum* [107].

Among PGPB, there are strains capable of synthesizing various antibiotics. Bacteria representing the genera *Bacillus, Pseudomonas*, or *Micromonospora* deserve special attention. The antibiotics produced by these bacteria have an inhibitory or killing effect on phytopathogens. In addition, it has been found that some of these compounds also have antiviral, anticancer, antioxidant, and cytotoxic properties [90,108]. The antibiotics produced by *Bacillus* include bacilysin, chlortetain, subtilin, bacillaene, or surfactin. Strains representing the genus *Pseudomonas* were found to have the ability to synthesize 2,4-diacetyl phloroglucinol (DAPG), pseudomonic acid, phenazine-1-carboxylic acid (PCA), pyoluteorin, pyrrolnitrin, or oomycin A [90,108].

PGPB have the ability to prevent infections caused by phytopathogens by activating immune mechanisms in the plant, i.e., the induced systemic resistance (ISR) mechanism. Contact of a plant host with a PGPB strain plays a key role in ISR induction. In the process of induced systemic immunity, unlike systemic acquired resistance (SAR), there is no synthesis of salicylic acid (SA) and production of defense proteins (PR). Jasmonic acid (JA) and ethylene are signaling molecules that play a major role in ISR. The plant defense process is controlled by NPR1 regulatory proteins. In the case of bacteria, lipopolysaccharide (LPS), siderophores, volatile compounds, such as 2,3-butanediol, or flagelles, can be signals causing ISR in a plant [108,109]. Microorganisms capable of inducing ISR include, inter alia, bacteria of the genus *Bacillus* spp. It has been shown that inoculation of cotton (*Gossypium hirsutum*) with *Bacillus* spp. strains protected the plant against *Spodoptera exigua* [110]. *Serratia liquefaciens* MG1 and *Pseudomonas putida* IsoF were shown to trigger induced systemic resistance (ISR) against *Alternaria alternata* in tomato (*S. lycopersicum*) [111].

Research on PGPB shows that some of these microorganisms have the ability to form a biofilm on plant roots. The biofilm provides the plant host with protection from external stressors, thus promoting crop growth and quality. It can constitute a physical barrier protecting the plant against the action of phytopathogens. The study also showed that the adhesion of bacteria to the roots may affect the induced systemic resistance in plants [112]. Literature data show higher IAA production, phosphate solubilization, siderophore production, and ammonia production by biofilm-forming microorganisms such as *Pseudomonas, Trichoderma, Bradyrhizobium*, and *Penicillium*, compared to microorganisms in the form of plankton [113,114,115].

Also, other mechanisms determined by the presence of endophytes in the host plant tissues are involved in overcoming the stress induced by the presence of xenobiotics in the plant growth environment. Bacteria metabolize aromatic pollutants in both the presence and absence of oxygen, but the former is more common. In aerobic metabolism, the two major products of the central degradation pathway are catechol and protocatechuate, which are then processed by ring-cleaving in the peripheral *ortho* or *metha* pathways into intermediates easily incorporated into the tricarboxylic acids pathway (TCA) in bacterial cells and by plants through the conjugation reactions with endogenous plant compounds. In this way, endophytes on the one hand reduce the content of contaminants in plant tissues and, on the other hand, can supply plants with nutrients [16,34,116]. In order for organic pollutants, which are usually hydrophobic in nature, to be metabolized by both bacteria and plants, they must be taken up by the cells of both organisms. However, the low bioavailability of these compounds usually makes their uptake difficult. Therefore, endophytes, which produce extracellular substances generally called biosurfactants reducing the surface tension at the interface of two phases, facilitate the transport of these compounds across biological membranes. Many such glycolipid or lipopeptide substances have been identified in endophytic bacteria [27]. It has also been shown that biosurfactants can exert a beneficial effect on plant growth also in non-stress conditions, which indicates that they can be new bioelicitors stimulating plant growth and development not considered so far [117]. In a study conducted by Marchut-Mikołajczyk et al. [118], glycolipids derived from *Bacillus pumilus* 2A isolated from *Chelidonium majus* L. herb significantly improved the growth of bean, radish, and beetroot.

In addition, other mechanisms consist in the promotion of metabolic gene expression and regulation of plant enzyme system activity. Recent findings indicate that the presence of endophytes can influence the activity of host-plant genes and enzymes, which can regulate the processes of pollutant metabolism and lead to an increase in the effectiveness of phytoremediation [119,120].

## 4. Methods of Selecting Endophytic Strains with Biodegradation Potential

Many culturable xenobiotics-degrading bacteria have been isolated based on their capacity to use organic pollutants as their unique energy and carbon sources; hence, studies on endophytic strains with biodegradation potential have mainly focused on the biochemical pathways until recently. However, culture-based identification of diverse microbial populations in a contaminated environment is a challenging task limited to fast-growing microbial diversity. Moreover, only 1% of the total microbial communities can be cultivated. Therefore, the acquisition of such isolates needs to be enhanced through the integration of the community-based approach with biodegradation pathways. However, few studies have implemented this concept to date, and most strains for application in consortia are still chosen in culture-based in vitro assays [19,36,50]. Thus, to improve the selection of candidates for such bacterial communities, omics-based technologies can be applied. The omics techniques, including metagenomics, proteomics, transcriptomics, and metabolomics, can be successfully employed for the characterization of pollutant-degrading endophytes, their metabolic machinery, novel proteins, and catabolic genes involved in the degradation processes to provide a deeper understanding of the complex reactions within such populations and unravel the complete microbial diversity living within plants [121].

Generally, the type and abundance of the endophytic community of a plant is strongly influenced by the selective pressure of contaminants. However, it is the plants that mostly “select” their microbiome in order to have beneficial colonizers [10,122]. Analyses of endophytes from stems of *Achillea millefolium*, *Solidago canadensis*, *Trifolium aureum*, and *Dactylis glomerata* plants derived from soil that was highly contaminated by hydrocarbons demonstrated that the class *Actinobacteria* was the dominant group among culturable isolates in all species, except *S. canadensis*, in which *Gammaproteobacteria* were the most abundant. 16S-based terminal restriction fragment length polymorphism analysis (TRFLP) data showed that endophytic bacterial communities were host-species specific [122]. Also, NGS sequencing of endophytic bacterial communities of two different types of grass grown in PHC-contaminated saline soil revealed higher diversity of the community in *Chloris virgata* than in the community of *Phragmites australis* [123]. These indicates that personalized bacterial cultures acting as bioinoculators should be assigned to specified plant systems. Moreover, it was demonstrated that accumulation of PAHs in plants providing a beneficial environment for xenobiotic-degrading endophytes resulted in the displacement of indigenous endophytic strains, which shows a limited capacity of plant tissues [10,26].

The distribution of endophytes within a given plant is also not uniform. This is mostly related to the structure of vascular tissues and the K*_OW_* value of the contaminant. The analysis of the microbiomes of roots and shoots of PHC-treated willow cultivars showed that distinct selected taxonomic classes are favored in these two niches.

*Gammaproteobacteria* and *Alphaproteobacteria* endophytes were more abundant in roots, while *Betaproteobacteria* were predominant in stems [122]. In turn, the abundance analysis indicated that the exposure of ryegrass to increasing concentrations of phenanthrene and pyrene positively correlated with the numbers of endophytic bacteria and negatively correlated with the richness of these communities in roots, while no such relationship was confirmed for shoots [25]. Similar data related to endophyte biodiversity in wheat depending on the phenanthrene contamination level were reported by Liu et al. [26]. Also, the endophytic bacterial community structure and function in stems of herbaceous plants from PHC-contaminated and non-contaminated sites showed no significant differences in the abundance of culturable endophytes and TRFLP phylotype richness [124]. All these findings might be explained by the fact that PAH accumulation is higher in the root compartment, where changes will be more severe. Particular xenobiotics have different physicochemical features, which should be kept in mind when designing pollution-degrading consortia.

When introduced into plants other than their adapted host, some endophytic microbes can cause growth repression and in extreme cases death in seedlings. Thus, modern molecular-based techniques can also be applied to evaluate the impact of inoculation on the structure of the indigenous endophytic bacterial community, i.e., endobiome interference [125]. Using the NGS technology with the Illumina platform, [26] established that the structure of the wheat bacterial community changed not only in a contamination level-dependent manner but also after the inoculation with *Massilia* sp. Pn2, originally derived from amur foxtail (*Alopecurus aequalis* Sobol). As a result, an increase in the diversity and richness of endophytic bacteria were observed [26].

Since the type of soil and organic contaminations contained therein affect the population characteristics of endophytic bacteria and selectively enhance the prevalence of endophytes containing genes encoding for enzymes of anabolic pathways for xenobiotics, these genes can be used for quick recognition of the biodegradative potential of the community examined. In their gene probe analysis, Siciliano et al. [10] showed a positive correlation between the soil creosote concentration and the number of detected endophytes with catabolic genes *alkB* and *ndoB* (gene for naphthalene dioxygenase) inside roots of *Festuca arundinacea*. Similarly, the numbers of *xylE*-positive (catechol-2,3-dioxygenase-positive) endophytic bacteria increased in *F. arundinacea* grown in the presence of nitroaromatics [10]. In turn, PCR analysis of culturable PHC-degrading endophytic bacteria obtained from *Lotus corniculatus* L. and *Oenothera biennis* L. derived from a long-term PHC-polluted site revealed that equal amounts of the isolates (about 40%) had the CYP153 gene involved in the degradation of alkanes and the a*cdS* gene encoding ACC deaminase, indicating a possibility for the future to choose between multiple strains for biotechnological purposes [24]. In comprehensive studies including genomic, physiological, metaproteomic, and in silico analysis, Macchi et al. [126] reconstructed a metabolic network showing the potential role of strains *Sphingobium* sp. AM, *Klebsiella aerogenes* B, *Pseudomonas* sp. Bc-h and T, *Burkholderia* sp. Bk, and *Inquilinus limosus* Inq in total phenanthrene degradation as a synthetic consortium with synergistic action. This approach was proposed for designing and optimization of bacterial consortia to tackle the bioremediation of complex environmental pollutants.

Functional metagenomics was also shown to be useful for improving the isolation of endophytic bacteria with specified metabolic features, which could be modulated depending on different compositions of plant media and culture conditions. An assessment of the metabolic potential of endophytic microbial communities in two rice cultivars cultured gnotobiotically pertaining to PGP traits, i.e., nitrogen fixation, phosphate solubilization, ACC deaminase production, ammonia production, amylase production, siderophore production, and IAA production, based on the relative abundance of bacterial OTUs was carried out with the use of PICRUSt-mediated analysis. Functional annotation revealed the presence of several PGP trait-related genes having variable relative abundance in tissue-specific and genotype-dependent manners. An in-silico study also documented a higher abundance of certain genes in the same biochemical pathways, such as nitrogen fixation, phosphate solubilization, and IAA production, implying their crucial roles in the biosynthesis of metabolites leading to PGP [6].

Research of the PGP effect of endophytes on host plants is mostly based on the biosynthesis of secondary metabolites by endophytes. However, the exact mechanisms by which these endophytes exert their bioactivity are largely unknown; hence, induction of plant gene expression in the presence of endophytes can provide clues about their effects on the host plant. To explore the impact of endosymbiotic bacteria and fungi on gene expression, metabolism, and other physiological aspects essential in conferring resistance against biotic and abiotic stresses in the willow, tripartite metatranscriptomics of a root microbiome responding to soil contamination was performed. The number of contigs functionally related to aromatic pollutant metabolism, e.g., biosurfactant production, toluene tolerance, and degradation of alkane sulfonates, were upregulated, suggesting that the bacterial species present in association with willow roots actively expresses an enzyme suite capable of degrading hydrocarbons present in contaminated soil and may play an important role within any tripartite interaction of mutual benefit. The study also revealed that bacterial gene expression included the apparatus necessary for biofilm interaction and direct reduction of contamination stress [127]. These findings emphasize the importance of the search for PGP-isolates capable to alleviate abiotic stress in parallel to catabolic pathways for their potential use in supported phytoremediation.

The chosen examples of culture-independent studies on endophytes with the potential to use in bacteria-assisted phytoremediation are included in Table 2.

## 5. Conclusions

The current methods targeted at the reduction of POP contaminations in the environment are insufficient. One of the conventional remediation techniques for rejuvenating land and aquatic systems polluted with anthropogenic contaminants is phytoremediation through the cultivation of specific crops. To enhance the treatment efficiency and the range of target POPs, the microbial-assisted phytoremediation approach can be applied. However, most bacteria are non-culturable; hence, the limited knowledge of the mechanisms underlying the interactions between plants and xenobiotic-degrading and/or PGP endophytic bacteria, which hampers the application of endophytes in phytoremediation. Thus, the exploration of structural and functional aspects of microbial communities with the more advanced and multifaceted omics approach can help to break the barrier. This modern technology can also be helpful in designing appropriate bacterial consortia for the removal of POPs. Such knowledge can be extended to address such compounds as hormones, PCPs, or less common PHA derivatives that have not been recognized as public health hazards so far, which may contribute to the fast development of methods for removal of these micropollutants. However, it is a prerequisite to culturing xenobiotic-degrading endophytic bacteria for their application in sustainable agriculture. This review article discusses the biodegradation potential of endophytic bacteria and the mechanisms they use for this purpose to indicate features that should be taken into account in designing communities used to enhance the phytoremediation effect.

## Figures and Tables

**Table 1:** Examples of interactions of PGP-endophytes with their host plants allowing for removal of selected xenobiotics.

Endophytic Strain	Host Plant	Degraded Xenobiotic	PGP-Activity	References
*Pseudomonas aeruginosa* L10	roots *Phragmites australis*	C_10_—C_26_ *n*-alkanes, naphthalene, phenanthrene, pyrene	Siderophore, biosurfactant and IAA production, ACC activity	[27]
*Bacillus safensis* ZY16	roots *Chloris virgate* Sw	C_12_—C_32_ *n*-alkanes, naphthalene, phenanthrene, pyrene	Siderophore, biosurfactant and IAA production, ACC activityphosphate solubilization	[17]
*Enterobacter* sp. 12J1	*Allium macrostemon* Bunge	pyrene	Siderophore, IAA production, phosphate solubilization	[30]
*Bacillus* sp. SBER3	*Populus deltoides*	anthracene, naphthalene, benzene, toluene and xylene	Siderophore, IAA production, ACC activity, phosphate solubilization, biocontrol activity	[20]
*Burkholderia* sp. PsJN	*Onion roots*	Textile effluent	ACC activity	[89]
*Microbacterium arborescens* TYSI04	shoot *T. domingensis*			[49]
*Bacillus pumilus* PIRI30	roots *Pistia*			[49]
*Pseudomonas aeruginosa* RRA, *Bacillus megaterium* RRB, *Sphingobacterium siyangensis* RSA, *Stenotrophomonas pavanii* RSB and *Curtobacterium plantarum* RSC	*Oryza sativa* L.	chlorpyrifos	Siderophore, IAA production, ACC activity, phosphate solubilization	[68]
*Pantoea dispersa*	roots *Dracena sanderiana*	Bisphenol A	IAA production, ROS scavenging	[58]
*Streptomyces griseorubiginosus* strains DS24 and DS4	roots *Miscanthus × giganteus*	diclofenac and sulfamethoxazole	Siderophore, IAA production	[75]

**Table 2:** Examples of culture-independent analyses of endophytes communities with potential for bioremediation.

“Omics”—Based System	Used Technics	Source of Bacteria	Pollutant for Degradation	References
metagenomics	TRFLP of PCR-amplified 16S rRNA gene fragments obtained with restriction enzymes *PvuII* and *MscI*	*Achillea millefolium* *Dactylis glomerata* *Trifolium aureum* *Solidago canadensis*	PHCs	[122]
	16S rRNA gene amplicon sequencing	*Phragmites australis* *Chloris virgata*	PHCs with saline	[123]
functional metagenomics	*alkB, ndoB*, and *ntdA* genes probe analysis	*Festuca arundinacea* *Trifolium fragiferum*	nitroaromatics	[10]
proteomics	nanoLC-MS/MS with XCalibur 3.0.63 software	synthetic consortium	phenanthrene	[126]
